# Exome sequencing in individuals with cardiovascular laterality defects identifies potential candidate genes

**DOI:** 10.1038/s41431-022-01100-2

**Published:** 2022-04-26

**Authors:** Katinka Breuer, Korbinian M. Riedhammer, Nicole Müller, Birthe Schaidinger, Gregor Dombrowsky, Sven Dittrich, Susanne Zeidler, Ulrike M. M. Bauer, Dominik S. Westphal, Thomas Meitinger, Tikam Chand Dakal, Marc-Phillip Hitz, Johannes Breuer, Heiko Reutter, Alina C. Hilger, Julia Hoefele

**Affiliations:** 1 Institute of Human Genetics, University Hospital of Bonn, Bonn, Germany; 2 Department of Pediatric Cardiology, Pediatric Heart Center, University Hospital of Bonn, Bonn, Germany; 3 Institute of Human Genetics, Klinikum rechts der Isar, School of Medicine, Technical University of Munich, Munich, Germany; 4 Department of Nephrology, Klinikum rechts der Isar, School of Medicine, Technical University of Munich, Munich, Germany; 5 Department of Congenital Heart Disease and Pediatric Cardiology, University Hospital of Schleswig-Holstein, Kiel, Germany; 6 Department of Pediatric Cardiology, University of Erlangen-Nürnberg, Erlangen, Germany; 7 Pediatric Department, Asklepios clinics, Sankt Augustin, Germany; 8 Competence Network for Congenital Heart Defects & National Register for Congenital Heart Defects, German Center for Cardiovascular Research (DZHK), Berlin, Germany; 9 DZHK (German Center for Cardiovascular Research), partner site Munich Heart Alliance, Berlin, Germany; 10 Department of Internal Medicine I, Klinikum rechts der Isar, School of Medicine, Technical University of Munich, Munich, Germany; 11 Department of Biotechnology, Mohanlal Sukhadia University Udaipur, Udaipur, Rajasthan India; 12 DZHK (German Centre for Cardiovascular Research) Partner Site, Kiel, Germany; 13 Division of Neonatology and Pediatric Intensive Care Medicine, Department of Pediatrics and Adolescent Medicine, Friedrich-Alexander-University Erlangen-Nürnberg, Erlangen, Germany

**Keywords:** Disease genetics, Genetic counselling, Biological sciences

## Abstract

The birth prevalence of laterality defects is about 1.1/10,000 comprising different phenotypes ranging from situs inversus totalis to heterotaxy, mostly associated with complex congenital heart defects (CHD) and situs abnormalities such as intestinal malrotation, biliary atresia, asplenia, or polysplenia. A proportion of laterality defects arise in the context of primary ciliary dyskinesia (PCD) accompanied by respiratory symptoms or infertility. In this study, exome sequencing (ES) was performed in 14 case-parent trios/quattros with clinical exclusion of PCD prior to analysis. Moreover, all cases and parents underwent detailed clinical phenotyping including physical examination, echocardiography by a skilled paediatric cardiologist and abdominal ultrasound examinations not to miss mildly affected individuals. Subsequent survey of the exome data comprised filtering for monoallelic *de novo*, rare biallelic, and X-linked recessive variants. In two families, rare variants of uncertain significance (VUS) in *PKD1L1* and *ZIC3* were identified. Both genes have been associated with laterality defects. In two of the remaining families, biallelic variants in *LMBRD1* and *DNAH17*, respectively, were prioritized. In another family, an ultra-rare *de novo* variant in *WDR47* was found. Extensive exome survey of 2,109 single exomes of individuals with situs inversus totalis, heterotaxy, or isolated CHD identified two individuals with novel monoallelic variants in *WDR47*, but no further individuals with biallelic variants in *DNAH17* or *LMBRD1*. Overall, ES of 14 case-parent trios/quattros with cardiovascular laterality defects identified rare VUS in two families in known disease-associated genes *PKD1L1* and *ZIC3* and suggests *DNAH17*, *LMBRD1*, and *WDR47* as potential genes involved in laterality defects.

## Introduction

Laterality defects, including situs inversus totalis and heterotaxy, are rare congenital anomalies of embryonic left-right axis patterning with a reported birth prevalence of about 1.1 cases per 10,000 live births [1]. While pre- and postnatal diagnosis and treatment of laterality defects, especially cardiovascular defects, has improved over time, the disease remains challenging for clinicians [2]. The exception is represented by individuals with situs inversus totalis in whom visceral organs are completely mirrored regarding their left-right axis patterning but are functionally normal. Those individuals, for which treatment remains challenging, are individuals with heterotaxy and situs ambiguus. 90% of these present with complex congenital heart defects (CHD) and situs abnormalities of their abdominal or thoracic organs [3, 4]. Interestingly, 3–7% of all isolated CHD have been suggested to arise from abnormal embryonic left-right axis patterning comprising double outlet right ventricle (DORV), atrioventricular canal defect (AVCD), or transposition of the great arteries (TGA) [3, 4]. The genetic background of laterality defects is heterogeneous with autosomal dominant, autosomal recessive, and X-linked inheritance [5, 6]. Laterality defects occur isolated or as part of complex genetic syndromes such as primary ciliary dyskinesia (PCD) syndromes. Hitherto, about 40 genes have been associated with the formation of laterality defects without PCD, comprising for example *LEFTY1*, *LEFTY2*, *NODAL*, and *PKD2*. Overall, a genetic cause can be identified in about 20% of all cases. The NODAL signaling as well as the SHH (sonic hedgehog) signaling cascade play an important role in ciliary assembly and function [3]. In order to prioritize genes previously not linked to monogenic laterality defects (“candidate genes”), exome sequencing of 14 unrelated families with cardiovascular laterality defects without PCD was performed.

## Methods

### Individuals and DNA isolation

The study was conducted in accordance with the Declaration of Helsinki, and ethical approval was obtained from the local ethic committee (Lfd Nr. 141/15). Each participating family provided written informed consent. Since family members of the index case might present with clinically unapparent features of the laterality defect spectrum, all family members included received a thorough clinical exam and ultrasound studies by two pediatric cardiologists in order to assess the affection status of all study participants. In total, 14 unrelated families affected by cardiovascular laterality defects were enrolled in the discovery cohort of this study. DNA was extracted from blood or saliva samples using the Chemagic Magnetic Seperation Module I (Perkin Elmer Chemagen Technologies GmbH, Baesweiler, Germany) or the Oragene DNA self-collection kit (following the Oragene TM DNA Purification Protocol for saliva samples).

### Exome sequencing

Exome sequencing was performed in 13 case-parent trios and one case-parent -quattro (two affected siblings and parents) using a Sure Select Human All Exon 60 Mb V6 Kit (Agilent) and a HiSeq4000 (Illumina) as previously described [7]. Reads were aligned to the human reference genome (UCSC Genome Browser build hg19) using Burrows-Wheeler Aligner (v.0.7.5a).

All exomes were initially analyzed in a diagnostic intent for disease-causing variants in known disease-associated genes. Detection of single-nucleotide variants and small insertions and deletions (indels) was performed with SAMtools (version 0.1.19). ExomeDepth was used for the detection of copy number variations (CNVs). A noise threshold of 2.5 was accepted for diagnostic analysis [8]. For every analysis, in a first step, a search for nonsynonymous variants (i.e., nonsense, frameshift, canonical splice site, missense, initiation codon, stoploss variants, and indels) and CNVs was conducted. If no disease-causing variant(s) could be detected the search was extended to near-splice, synonymous, intronic, and untranslated-region (UTR) variants (provided that there was coverage). Variants were visualized with the Integrative Genomics Viewer (IGV, https://software.broadinstitute.org/software/igv/). Rating of variants was done according to American College of Medical Genetics (ACMG) guidelines and current amendments [9–12]. For the analysis of *de novo*, autosomal dominant and mitochondrial variants, only variants with a minor allele frequency of less than 0.1% in the in-house database of over 22,000 exomes (“Munich Exome Server”) were considered. For the analysis of autosomal recessive and X-linked variants (homozygous, hemizygous, or putative compound heterozygous) only variants with a minor allele frequency of less than 1% were considered. Furthermore, variants were compared to publicly available databases such as the Genome Aggregation Database (gnomAD, v2.1.1). To confirm if a variant was already published a data search for the respective variant was performed in PubMed, ClinVar, and the Human Gene Mutation Database (HGMD®). CNVs were also visualized with IGV to check for sufficient coverage of the inspected region and plausibility of the CNV. CNVs were compared with publicly available control databases like gnomAD, the Database of Genomic Variants (DGV, http://dgv.tcag.ca/dgv/app/home) and databases for pathogenic CNVs like DECIPHER (https://decipher.sanger.ac.uk/) and the aforementioned databases PubMed, ClinVar and HGMD®. Likely pathogenic and pathogenic variants as per ACMG are summarized as “disease-causing variant” in the text.

Cases, in which no disease-causing variant in known disease-associated genes could be identified, were investigated in order to prioritize variants in potential candidate genes. The same analysis strategy and MAF thresholds as in the diagnostic setting were employed (see above). Potentially relevant variants were checked for nucleotide/amino acid conservation (UCSC browser; https://genome.ucsc.edu/), in silico prediction of deleteriousness using Sorting Intolerant From Tolerant (SIFT; ≤0.05), PolyPhen-2 (pph2; 0.85–1.0) and Combined Annotation Dependent Depletion (CADD; ≥ 20). gnomAD constraint metrics were also used for gene assessment: A loss-of-function variant observed/expected ratio (depending on gene and sample size) with a loss-of-function observed/expected upper-bound fraction (LOEUF) <0.35 indicates that a gene is haploinsufficient; that is, heterozygous LoF variants (with expected NMD) will cause disease [13]. For missense variants, a Z-score >3.09 indicates selection against missense variants in the respective gene [14]. To look for functional and KO mouse data, a PubMed and Mouse Genome Informatics (MGI; http://www.informatics.jax.org/) search was conducted. Note that a lack of functional/mouse data was not a reason to exclude variants.

Confirmation of identified variants in the index and the relatives was carried out by Sanger sequencing.

Variants, which are not listed in gnomAD v.2.1.1, are called “novel” throughout the manuscript.

### Screening of candidate genes

In collaboration with the German Competence Network for Congenital Heart Defects and the Institute of Human Genetics at the University of Kiel, single exomes of 2,109 individuals with situs inversus totalis, heterotaxy, or isolated CHD were surveyed for rare variants in newly identified candidate genes. These samples include a subset from a previously published study as well as additional internal unpublished cases [15]. Filtering of exome datasets was performed analogous to the above-described criteria. Prioritized variants were confirmed using Sanger sequencing.

### Cilia diagnostics

Cilia diagnostics in individual HET22_501 were done by light microscopy of respiratory epithelial cells preserved by trans-nasal brushing and by nasal nitric oxide (nNO) measurement with NIOX Vero.

### Structural protein modelling of DNAH17

Three-dimensional protein structural models for DNAH17 were built using I-TASSER (Iterative Threading ASSEmbly Refinement (https://zhanglab.ccmb.med.umich.edu/I-TASSER/). Since, the server cannot handle large protein sequence, for the prediction of DNAH17 amino acid changes the sequence was trimmed to 120 amino acid sequences including mutated site and then the sequence was subjected to I-TASSER based modeling. The structural comparison between wild-type and mutated protein was done in UCSF Chimera after superimposing the structure of mutant onto the wild structure using SuperPose using default parameters (superpose.wishartlab.com).

## Results

### Phenotype of individuals enrolled in this study

Two of the 14 individuals showed situs inversus totalis with patent foramen ovale (PFO). 10 individuals were born with heterotaxy and a CHD that required treatment and two individuals were born with heterotaxy accompanied by vascular malformations. None of the affected individuals presented with clinical signs of PCD comprising chronic respiratory tract infections or infertility. Ultrasound studies of family members revealed no phenotype in terms of laterality defects, but one father showed on one side double kidney and one brother had renal agenesis.

### Exome sequencing

Filtering of exome datasets in all affected individuals for possible disease-causing variants in known disease-associated genes for laterality defects revealed variants of uncertain significance (VUS) in *PKD1L1* and *ZIC3*. In the remaining individuals, variants in three candidate genes namely *LMBRD1*, *DNAH17*, and *WDR47* were identified.

### Identification of variants of uncertain significance in the known disease-associated genes *PKD1L1* and *ZIC3*

In a male individual (HET25_501; Table 1) with heterotaxy comprising left sided inferior vena cava, joining right atrium, midline positioned liver, accessory spleen, and membranous duodenal stenosis, a compound heterozygous VUS in *PKD1L1* coding for Polycystin 1-Like 1 was found. The parents were heterozygous carriers of these variants. The maternally inherited variant in exon 36 (NM_138295.3:c.5660C>T, p.(Thr1887Met), rs369417620; ACMG rating: PM1_supp PM2) has a MAF of 0.00003719 and has not been reported in a homozygous state in gnomAD. *In-silico* prediction of this variant by pph2 and SIFT classified the variant as “probably damaging” and “tolerated” respectively. The CADD score was 19.8. The paternally inherited variant is an in-frame insertion of 15 base pairs (NM_138295.3:c.4019_4033dup, p.(Gln1344_Trp1345insSerSerCysAsnGln); ACMG rating: PM1_supp PM2 PM4) and not listed in gnomAD.

**Table 1:** Overview of individuals with variants of uncertain significance in the known disease-associated genes *PKD1L1* and *ZIC3*.

Family	HET9	HET25
**Origin**	Germany	Germany
**Consanguinity**	no	no
**Laterality defect**	situs inversus totalis with mesocardia, functional univentricular AVCD, TGA with pulmonary atresia, left aortic arch with truncus bicaroticus	heterotaxy with left sided IVC joining right atrium, midlined liver, accessory spleen
**Respiratory phenotype**	no	no
**Other features**	hypoplastic right fenestra ovalis, malformation right stapes	bronchial asthma, membranous duodenal stenosis, gastroesophageal reflux
**Ultrasound findings family**	brother: left sided renal agenesis	**–**
**Disease-associated gene**	*ZIC3*	*PKD1L1*
**Nucleotide change (with transcript number)**	NM_003413.3:c.1195C>T	NM_138295.3:c.5660C>T NM_138295:c.4019_4033dup
**Amino acid change**	p.(His399Tyr)	p.(Thr1887Met) p.(Gln1344_Trp1345insSerSerCysAsnGln)
ACMG rating*	PM1_supp PM2 PP3	PM1_supp PM2 PM1_supp PM2 PM4
**Zygosity**	hemizygous	compound heterozygous
**Inheritance**	maternal	maternal/paternal

*AVCD* Atrioventricular canal defect, *TGA* Transposition of great arteries, *IVC* Inferior vena cava; *[9–12].

In another male individual (HET9_501; Table 1), a hemizygous VUS in *ZIC3* (NM_003413.3:c.1195C>T, p.(His399Tyr); ACMG rating: PM1_supp PM2 PP3) coding for zinc finger protein ZIC 3 was identified. This individual presented with situs inversus totalis, mesocardia, functional univentricular AVCD, TGA with pulmonary atresia and left aortic arch with truncus bicaroticus. The variant was inherited from the healthy mother. A brother of the index presented with left sided renal agenesis. While laterality defects are usually not associated with unilateral renal agenesis, we Sanger sequenced the variant in his brother revealing a wild type sequence. The variant is located at an evolutionary conserved position within the zinc finger domain, a domain suspected to guide development of left-right asymmetry during embryogenesis [16]. *In-silico* prediction of this variant by pph2 and SIFT classifies the variant to be “probably damaging” respectively “damaging”. The CADD score was 28.9.

### Rare variants in *LMBRD1*, *DNAH17*, and *WDR47* might be linked to cardiovascular laterality defects

#### LMBRD1

**Fig. 1:**
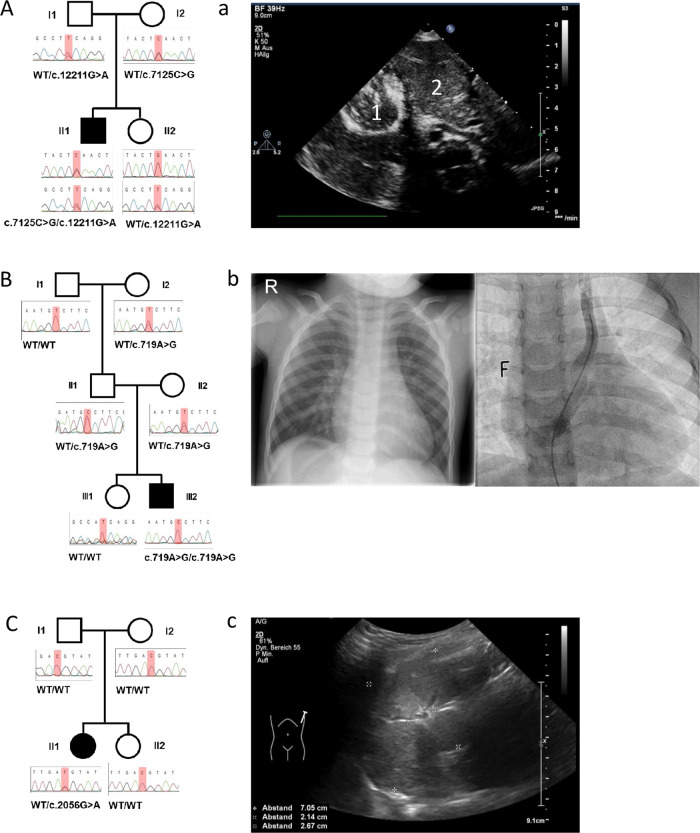
Clinical and genetic information on families HET22, HET11, and HET5. **A**, **a** Pedigree of HET22 with rare compound heterozygous variants in *DNAH17* (**A**) and phenotype of the index HET22_501: abdominal ultrasound with a right sided stomach (1) and a left sided liver (2) (**a**). **B**, **b** Pedigree of HET11 with a rare homozygous variant in *LMBRD1* (**B**) and phenotype of the index HET11_501: X-ray thorax with a right sided gastric bubble and intracardiac catheter picture with persistent left sided SVC (**b**). **C**, **c** Pedigree of HET5 with a rare heterozygous *de novo* variant in *WDR47* (**C**) and phenotype of the index HET5_501: abdominal ultrasound showing polysplenia (**c**).

In a male individual (HET11_501) with complex anomalies, a homozygous missense variant (NM_018368.3:c.719A>G, p.(Asp240Gly)) in *LMBRD1* (Table 2 and Fig. 1) was identified. The non-consanguineous parents were heterozygous carriers of the respective variant. *LMBRD1* encodes for lysosomal cobalamin transport escort protein LMBD1. *LMBRD1* has been associated with methylmalonic aciduria and homocystinuria (cblF disorder, OMIM #277380). The clinical phenotype is variable comprising small for gestational age, poor feeding, failure to thrive and developmental delay. Interestingly, 7 out of 15 children had CHD or laterality defects (Supplementary Table 1) [17–19]. In the present study, metabolic analysis of individual HET11_501 did not show any abnormalities. Electron microscopy of ciliated cells was normal. In silico prediction of the identified variant by pph2 and SIFT classified the variant as “probably damaging” and “tolerated” respectively. The CADD score was 24.9. In addition, the variant is not present in gnomAD. In collaboration with the German Competence Network for Congenital Heart Defects single exomes of 2,109 individuals with situs inversus totalis, heterotaxy, or isolated CHD were surveyed for putative variants in *LMBRD1*. This analysis did not identify any homozygous or compound heterozygous rare variants among these individuals in *LMBRD1*.

**Table 2:** Overview of individuals with rare variants in *LMBRD1*, *DNAH17*, and *WDR47*.

Family	HET22	HET11	HET5	EGAN00001389851	EGAN00001387735
**Origin**	Germany/Sri Lanka	Germany	Germany	Germany	Germany
**Consanguinity**	no	no	no	NA	NA
**Laterality defect**	situs inversus totalis with PFO	heterotaxy with bilateral SVC with bridging vein, left SVC outlet into dilated coronary sinus, left sided IVC, mitral valve stenosis, subaortical stenosis, pulmonary valve dysplasia, VSD, ASD, SVE, intestinal malrotation, duodenal atresia, right sided stomach, right sided polysplenia, left sided liver	heterotaxy with AVCD, vena azygos continuity, artery lusoria, truncus bicaroticus and polysplenia	discordant ventriculo-arterial connections	VSD
**Respiratory phenotype**	no	no	no	NA	NA
**Other features**	cilia diagnostics (light microscope): inconspicuous	bronchial asthma, cilia diagnostics (electron microscopy): inconspicuous metabolic diagnostic: inconspicuous	–	–	Talipes cavus equinovarus
**Ultrasound findings family**	–	father: left sided double kidney	–	NA	NA
**Gene**	*DNAH17*	*LMBRD1*	*WDR47*	*WDR47*	*WDR47*
**Nucleotide change (with transcript number)**	NM_173628.3:c.7125C>G NM_173628.3:c.12211G>A	NM_018368.3:c.719A>G	NM_014969.5:c.2056G>A	NM_014969.5:c.1208C>A	NM_014969.5:c.1378G>A
**Amino acid change**	p.(Phe2375Leu)p.(Glu4071Lys)	p.(Asp240Gly)	p.(Val686Ile)	p.(Pro403His)	p.(Val460Met)
**Zygosity**	compound heterozygous	homozygous	heterozygous	heterozygous	heterozygous
**Inheritance**	maternal/paternal	maternal/paternal	*de novo*	unknown	unknown

*NA* Not available, *PFO* Patent foramen ovale, *SVC* Superior vena cava, *IVC* Inferior vena Cava, *VSD* Ventricular septal defect, *ASD* Atrial septal defect, *SVE* Supraventricular extrasystoly, *AVCD* Atrioventricular canal defect.

#### DNAH17

Dynein axonemal heavy chain 17 (DNAH17) is a protein of the DNAH family of which four genes have already been associated with autosomal recessive PCD, situs inversus totalis or heterotaxy, namely *DNAH1*, *DNAH5*, *DNAH9*, and *DNAH11* [20–23]. Here, in a male individual (HET22_501) with situs inversus totalis with PFO, rare compound heterozygous missense variants in exon 46 and exon 75 in *DNAH17* (NM_173628.3:c.7125C>G, p.(Phe2375Leu), paternally inherited and NM_173628.3:c.12211G>A, p.(Glu4071Lys), maternally inherited) were found. The index did not show any signs of PCD. Light microscopy of ciliated cells of the respiration tract and nasal NO-testing with a NO-concentration of 322 ppb were normal. The variants were both located in a highly conserved position with a CADD score of 24.6 for p.(Phe2375Leu) and a CADD score of 32 for p.(Glu4071Lys). SIFT and pph2 predicted the variants to be “damaging” or “probably damaging”. The variant c.12211G>A is not listed in gnomAD. The variant c.7125C>G (rs751260682) has been described three times in 248,992 alleles in gnomAD (MAF of 0.00001205). Screening of all single exomes of 2109 individuals with situs inversus totalis, heterotaxy or isolated CHD detected no additional homozygous or compound heterozygous rare variants in *DNAH17*.

**Fig. 2:**
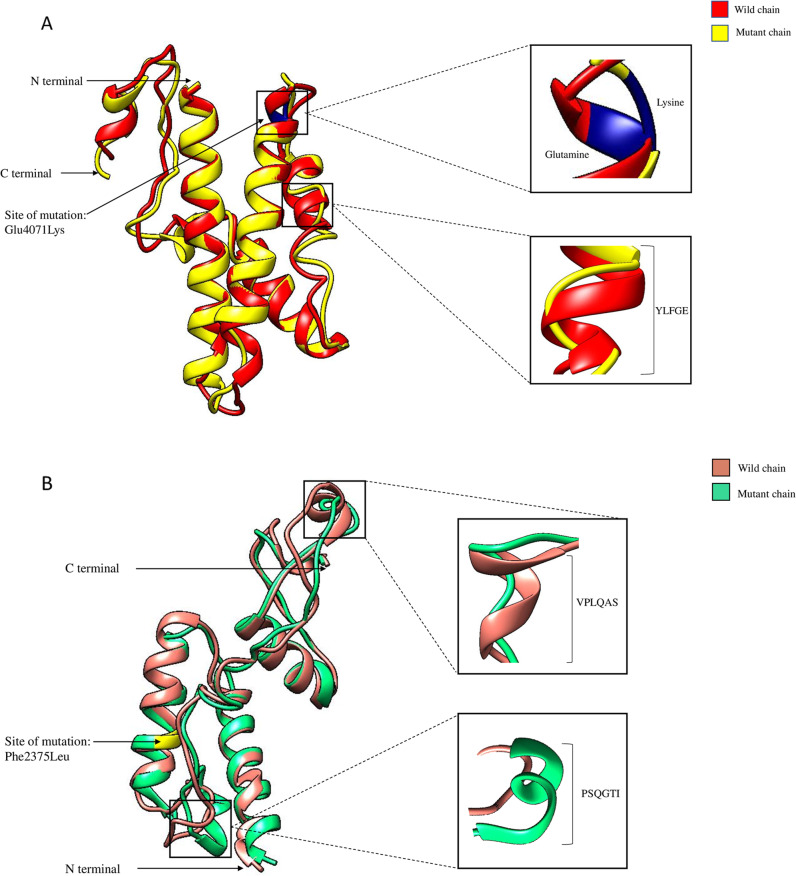
Structural modelling and in-silico analysis of DNAH17 protein. **A** Amino acid change from glutamine at position 4071 to lysine causing structural variation at the site of variant in DNAH17. The wild chain has formed helix whereas mutated chain formed a loop. Structural variation was found in the position 4083-4087 having amino acid sequence YLFGE. In the wild chain, the sequence has formed a helix whereas in the mutated chain it has formed a loop. **B** Amino acid change from phenylalanine at position 2375 to leucine causes no structural variation at the site of variant in DNAH17 and was found to be superimposed with the mutant chain. But, there was structural variation at positions 2389-2397 and 2422-2427. At position 2389-2397 containing amino acid residues PSQGTI forms loop in the wild chain whereas it formed helix in the mutated chain. At position 2422-2427 containing amino acid residues VPLQAS forms helix in the wild chain whereas it formed a loop in the mutated chain.

Structural modelling and *in-silico* analysis of DNAH17 protein (Fig. 2) showed that the variant at position c.12211 of *DNAH17* resulted in a substitution of p.(Glu4071Lys) with a RMSD value amounting to 1.61 Å at C-alpha carbon and 1.69 Å in the protein backbone. Also, the variant at position c.7125 resulted in a substitution of p.Phe2375Leu with a RMSD value amounting to 2.19 Å at C-alpha carbon and 2.23 Å in the protein backbone. Both variants generated structural variation in their predicted 3-D structure and neither superimposed due to variation, yet neither of the two variants affect any of the known functional domains of DNAH17 protein.

#### WDR47

A rare heterozygous *de novo* missense variant in *WDR47* (NM_014969.5:c.2056G>A, p.(Val686Ile)) was detected in a female individual (HET5_501) with heterotaxy including AVCD, vena azygos continuation, artery lusoria, truncus bicaroticus and polysplenia. The variant is listed two times in a heterozygous state in gnomAD (MAF of 0.000007109) and is in a highly conserved position with a CADD score of 22.9. Pph2 predicted the variant as “possibly damaging”, SIFT predicted it to be “tolerated”. Targeted Sanger sequencing in the parents and index confirmed the *de novo* status. Screening of all single exomes of 2,019 individuals with situs inversus totalis, heterotaxy, or isolated CHD detected two additional individuals with monoallelic novel missense variants in *WDR47*. One male individual (EGAN00001389851_501) presented with discordant ventriculo-arterial connections, the other female individual (EGAN00001387735_501) had a ventricular septal defect (VSD). Both variants (NM_014969.5:c.1208C>A, p.(Pro403His) and NM_014969.5:c.1378G>A, p.(Val460Met)) predicted to be “possibly damaging” by pph2 and “tolerated” by SIFT. The CADD scores were 23.7 and 25.2, respectively. Due to a lack of parental DNA samples, a statement concerning their *de novo* status cannot be made.

## Discussion

In two out of 14 families, variant filtering identified rare variants in the known disease-associated genes *PKD1L1* and *ZIC3*, which—due to limited evidence— need to be classified as VUS at the moment. In the remaining 12 families, no disease-causing variants in known disease-associated genes could be identified. In an investigation for genes previously not associated with laterality defects, biallelic rare variants in *LMBRD1* and *DNAH17* and a monoallelic rare variant in *WDR47* could be prioritized in unsolved cases suggesting these genes as possible new candidate genes for isolated cardiovascular laterality defects. Hence, exome sequencing of 14 case-parent trios/quattros identified rare variants of uncertain significance in two families in known disease-associated genes and identified three genes possibly associated with the development of cardiovascular laterality defects.

Variants in *PKD1L1* (Polycystin 1-Like 1) have been associated with failure in left-right patterning. So far, three cases with laterality defects and disease-causing variants in *PKD1L1* have been reported by Vetrini et al. and Le Fevre et al. [24, 25]. PKD1L1 forms a protein complex with PKD2 along primary cilia, which is able to sense nodal flow while responding to Ca^2+^ signal [26, 27]. Grimes et al. postulate that the sensation of nodal flow by PKD1L1 and PKD2 complex leads through a signal cascade to a removal of repression of Nodal signaling on the left side with a simultaneous maintenance of this repression on the right side [26]. Moreover, Grimes et al. and Field et al. showed in mouse models that point mutations in *Pkd1l1* lead to laterality defects even with different impacts on NODAL activity (bilateral NODAL activity versus complete absence of NODAL activity) whereas nodal ciliary structure, bending and rotation seem to be unaffected through defect or missing PKD1L1 [26, 27].

Variants in the gene *ZIC3* (Zic family, member 3) have been frequently associated with laterality defects as heterotaxy and corresponding CHD and have also been described to be associated with the VATER/VACTERL association [28–31]. Furthermore, *ZIC3* has been observed in individuals with isolated CHD [28]. Ware et al. postulate that approximately 1% of sporadic heterotaxy result from hemizygous variants in *ZIC3*, which lead to a disturbed left-right patterning through influencing the *nodal-related 1* and *Pitx2* signaling [16, 28]. In the present study, a novel variant at an evolutionary conserved region in *ZIC3* in an index with situs inversus totalis and CHD like AVCD, TGA with pulmonary atresia and left aortic arch was identified.

*LMBRD1* (LMBR1 domain containing protein 1) is associated with autosomal recessive methylmalonic aciduria and homocystinuria (cobalamin F type; cblF type) [17]. According to the literature, 15 cases with methylmalonic aciduria and homocystinuria, type cblF, have been reported. Age of onset and phenotype are very diverse comprising being small for gestational age, poor feeding, failure to thrive and developmental delay up to asymptomatic clinical course [17–19]. Interestingly Constantinou et al., Oladipo et al. and Rutsch et al. described, in seven out of 15 affected individuals, co-occurring laterality defects or CHD comprising dextrocardia, TGA, DORV, atrial septal defect (ASD) and VSD (Supplementary Table 1) [17–19]. Cobalamin F defect is caused by a defect in LMBD1, a lysosomal membrane protein, which is encoded by *LMBRD1*. LMBD1 transfers cobalamin from lysosome into cytoplasm [17]. If this process does not work, cobalamin accumulates in lysosomes and is not available for further vitamin B12 dependent metabolism through deficiency of the coenzymes methylcobalamin and adenosylcobalamin resulting in methylmalonic aciduria and homocystinuria [32, 33]. However, Buers et al. showed that Lmbd1 is also important for initiation of gastrulation and formation of mesodermal structures in early embryogenesis. *Nodal*, which encodes an important signal protein in formation of left-right axis, has been shown to be expressed slightly broader in whole-mount in-situ hybridization of *Lmbd1* deficient mice, but with his typical proximal-distal gradient [34]. Finally, *LMBRD1* could play a major role in establishing left-right axis during embryogenesis because of its impact on gastrulation and formation of mesodermal structures [34]. Interestingly, so far reported individuals with metabolic disorders in cobalamin metabolism had homozygous deletions or canonical splice site variants in *LMBRD1*. Here, a novel homozygous missense variant in *LMBRD1* in an individual with heterotaxy and CHD but without metabolic abnormalities could be found suggesting the possible involvement of *LMBRD1* in isolated cardiovascular laterality defects is independent of a metabolic disorder.

Dynein heavy chain is part of the outer dynein arms (ODA) in the motorprotein dynein. Different proteins of this group show high homology between each other. In the literature isolated male infertility and asthenozoospermia is described in individuals with variants in *DNAH17* [35]. The reported index is too young for male infertility testing and thus this symptom cannot be ruled out at the moment. Data from the Human Protein Atlas (http://www.proteinatlas.org) and additional GTex suggest that *DNAH17* is almost exclusively expressed in testis (no or only limited expression in internal organs; https://gtexportal.org/home/gene/DNAH17) and colocalizes with DNAH8 and α-tubulin along the axoneme of sperm flagellum [35, 36]. Whitfield et al. identified homozygous and compound heterozygous variants in *DNAH17* in individuals with isolated male infertility due to several morphological anomalies of sperm cells. Clinical examination of thoracic organs and ear, nose, throat by computerized tomography scan did not show any congenital malformations, situs inversus totalis or symptoms of PCD in these individuals. Hence, Whitfield et al. suggested that biallelic variants in *DNAH17* cause asthenzoospermia without other PCD related symptoms. These findings support our thesis that defects in DNAH17 do not harm cilia on respiratory cells [35]. Analogous, intact respiratory epithelial cells were found in our index on microscopic examination. Additionally, *DNAH17* has 80 coding exons (NM_173628.3) and shows extensive missense variation in gnomAD (z score = −3.13), so the identification of the compound heterozygous missense variants mentioned above could be coincidental and the variants could have no effect on protein function (in a monogenic way). In this context we performed *in-silico* protein modelling. Due to the results, one could speculate that the variants alter the overall conformation thus affecting the functional parts even though the variants themselves do not lie within a known domain. Taken together, this does not completely rule out that variants in *DNAH17* could possibly lead to dysfunction of nodal cilia through loss of ODAs or malfunction of ODAs leading to laterality defects without clinical trait of PCD but has to be further elucidated in larger cohorts. Since the additional survey of 2,109 single exomes of individuals with situs inversus totalis, heterotaxy, or isolated CHD did not identify biallelic homozygous or compound heterozygous variants in *DNAH17*, biallelic variants in *DNAH17* - if causative - might only be a rare cause of isolated situs inversus totalis with CHD.

Furthermore, missense variants in *WDR47* were found. One monoallelic *de novo* missense variant in *WDR47* in a female individual with cardiovascular heterotaxy and two novel monoallelic missense variants in *WDR47* in two individuals with isolated CHD were identified. *WDR47* is evolutionary highly conserved and encodes a protein called WD-repeat containing Protein 47, which is so far poorly understood, but known to regulate autophagy and microtubule dynamic instability via interactions with MAP8, a microtubule associated protein [37]. Kannan et al. showed that mice lacking *WDR47* evolve developmental disorders of the brain, notably microcephaly and corpus callosum defects [38]. However, in human so far no phenotype has been associated with variants in *WDR47* [38]. Expression analysis in adult mice showed that *Wdr47* is mainly expressed in different areas of brain with lower expression in liver, heart and lung [38]. During embryonic development in mice *Wdr47* is ubiquitous expressed with high levels of expression in lung and heart [39]. It is quite conceivable that - due to its relevance in microtubule dynamics and function— *WDR47* might play a role in embryonic left-right axis patterning [37]. Interestingly, *WDR16* of the WD40-repeat family has been associated with heterotaxy and situs inversus totalis [40]. Based on our findings and scientific reports from the literature, we suggest *WDR47* as a candidate gene for cardiovascular laterality defects and isolated CHDs.

Previously, the majority of genetic studies on laterality defects have focused on defects associated with PCD. In the present study, the focus was on cardiovascular laterality defects, without clinical signs of PCD. While our results suggest the identification of candidate genes, they are limited by the fact that we could not identify additional individuals with comparable defects and genetic findings that provide further support for *LMBRD1*, *DNAH17*, and *WDR47* as candidate genes. Concluding, according to MacArthur et al., the current evidence of the identified candidate genes concerning disease association is rather on the variant-level [41]. Further genetic and functional studies are therefore warranted to explore the contribution and role of *LMBRD1*, *DNAH17*, and *WDR47* in the formation of cardiovascular laterality defects. Moreover, one has to consider that besides monogenic mechanisms polygenic processes through complex pathways, which are part in forming laterality, can be causative for laterality defects and could explain the few cases with evidence of a monogenic disorder. Additionally, there is evidence on non-genetic factors like teratogenic exposure e.g., maternal diabetes [6].

## Supplementary information

Legends Supplementary Tables

Reported cases with variants in *LMBRD1* and a phenotype consistent with laterality defects.

Phenotypes of individuals without findings in WES.

## Data Availability

The data that support the findings of this study are available on request from the corresponding author.
