# Supplementary material for: Exome sequencing in individuals with cardiovascular laterality defects identifies potential candidate genes
Source: Eur J Hum Genet. 2022 Apr 26;30(8):946–54. doi: 10.1038/s41431-022-01100-2 (PMC9349204; doi:10.1038/s41431-022-01100-2)
Supplement: Supplementary file 1 — Legends Supplementary Tables [file 41431_2022_1100_MOESM1_ESM.pdf]

Supplementary Table 1: Reported cases with variants in *LMBRD1* and a phenotype consistent with laterality defects.

Supplementary Table 2: Phenotypes of individuals without findings in WES. ASD, atrial septal defect; AVCD, atrioventricular canal defect; ccTGA, congenitally corrected transposition of the great arteries; DORV, double outlet right ventricle; IVC, inferior vena cava; LSVC, left superior vena cava; PDA, patent ductus arteriosus; PFO, patent foramen ovale; VSD, ventricular septal defect; TGA, transposition of the great arteries.
